# Exploring core symptoms of alcohol withdrawal syndrome in alcohol use disorder patients: a network analysis approach

**DOI:** 10.3389/fpsyt.2024.1320248

**Published:** 2024-08-29

**Authors:** Guanghui Shen, Yu-Hsin Chen, Yuyu Wu, Huang Jiahui, Juan Fang, Tang Jiayi, Kang Yimin, Wei Wang, Yanlong Liu, Fan Wang, Li Chen

**Affiliations:** ^1^ Department of Behavioral Medicine, Wenzhou Seventh People’s Hospital, Wenzhou, China; ^2^ School of Mental Health, Wenzhou Medical University, Wenzhou, China; ^3^ The Affiliated Wenzhou Kangning Hospital, Wenzhou Medical University, Wenzhou, China; ^4^ Medical Neurobiology Lab, Inner Mongolia Medical University, Huhhot, China; ^5^ Beijing Hui-Long-Guan Hospital, Peking University, Beijing, China

**Keywords:** alcohol use disorder, alcohol withdrawal syndrome, network analysis, psychopathology, Lasso

## Abstract

**Background:**

Understanding the interplay between psychopathology of alcohol withdrawal syndrome (AWS) in alcohol use disorder (AUD) patients may improve the effectiveness of relapse interventions for AUD. Network theory of mental disorders assumes that mental disorders persist not of a common functional disorder, but from a sustained feedback loop between symptoms, thereby explaining the persistence of AWS and the high relapse rate of AUD. The current study aims to establish a network of AWS, identify its core symptoms and find the bridges between the symptoms which are intervention target to relieve the AWS and break the self-maintaining cycle of AUD.

**Methods:**

Graphical lasso network were constructed using psychological symptoms of 553 AUD patients. Global network structure, centrality indices, cluster coefficient, and bridge symptom were used to identify the core symptoms of the AWS network and the transmission pathways between different symptom clusters.

**Results:**

The results revealed that: (1) AWS constitutes a stable symptom network with a stability coefficient (CS) of 0.21-0.75. (2) Anger (Strength = 1.52) and hostility (Strength = 0.84) emerged as the core symptom in the AWS network with the highest centrality and low clustering coefficient. (3) Hostility mediates aggression and anxiety; anger mediates aggression and impulsivity in AWS network respectively.

**Conclusions:**

Anger and hostility may be considered the best intervention targets for researching and treating AWS. Hostility and anxiety, anger and impulsiveness are independent but related dimensions, suggesting that different neurobiological bases may be involved in withdrawal symptoms, which play a similar role in withdrawal syndrome.

## 1 Introduction

Alcohol is a widely used and harmful substance globally, with over two billion people using it and 283 million people experiencing an alcohol use disorder (AUD) (1, 2). The most effective therapy for alcoholism and related problems is alcohol withdrawal, but abruptly ceasing alcohol use often leads to alcohol withdrawal syndrome (AWS) (3). AWS is a prevalent health issue among individuals with AUD. It is characterized by hyperactivity of the central nervous system and autonomic nervous system (4). Approximately more than half of hospitalized patients with AUD and 35% of community individuals with AUD are at risk of developing AWS (5). While the physiological symptoms of acute alcohol withdrawal typically subside within a few days, certain dysregulations persist, such as blunted hypothalamic-pituitary-adrenal axis responsiveness (6) and reduced basal levels of circulating corticosteroids (7). Persistent dysregulations are thought to contribute to the mood and behavioral symptoms of AUD (8–10) and play a significant role in maintaining alcohol consumption and developing AUD. Negative affect and behaviors during alcohol withdrawal can lead individuals to drink in order to cope with dysphoria and negative emotions, creating a self-perpetuating addictive behavior cycle (AUD → Withdrawal → AWS → Drinking to Cope → AUD) that contributes to relapse and the continuous cycle of alcohol withdrawal (11, 12). This cycle helps explain why conventional interventions often limited success have, with relapse occurring in up to 80% of patients with severe AUD. Therefore, effectively managing AWS is crucial for breaking the self-perpetuating cycle of AUD (13).

AWS is not caused by a single mechanism but rather by multiple mechanisms (14, 15). AWS is characterized by various symptoms, including irritability, dysphoric mood, and physical symptoms such as headaches, seizures, and tremors (14–16). However, previous research has often focused on studying individual symptoms rather than considering them as part of a larger set of symptoms. The approach of focusing on a single symptom neglects the broader mental disorder and the interconnectedness and propagation of symptoms in mental disorders (17). Additionally, pharmacological treatments for AWS such as benzodiazepines and anticonvulsants attempt to restore balance by enhancing GABAergic inhibition. However, these medications only provide temporary symptomatic relief and do not fully address the underlying neurobiological changes from chronic alcohol exposure (18). Alcohol withdrawal symptoms frequently reemerge after discharge when medications are stopped, putting patients at high risk for relapse and recurrence of AUD (19). While medications can help manage acute withdrawal episodes, they do not provide a cure or long-term solution. This emphasizes the necessity for an enhanced comprehension of AWS mechanisms and symptom interactions develop more targeted and effective interventions that tackle the underlying causes.

Recently, the network theory and approach have been used to understand the interactions between symptoms and psychopathology in mental disorders. The Network Theory of Mental Disorders (NTMD) suggests that mental disorders do not result from a central functional disorder but rather arise from long-term, sustained feedback loops formed by the mutual influence of multiple mental symptoms (20). When a set of symptoms in a network structure are closely connected, they tend to synchronize. This complex system of mutually reinforcing dependencies, which can be modelled as edges within the network. This network consists of symptom nodes that activate and deactivate each other over time (20, 21). The concept of symptom networks has been explored in various studies (22). Furthermore, symptoms of mental disorders may have direct or indirect effect on each other and may have genetic, mental, and psychological bases (23). Therefore, comprehending the interactions and interconnectedness between different symptoms of AWS may aid in more effective and efficient relief or prevention of withdrawal symptoms. The network approach provides a powerful method for analyzing and visualizing the complex relationships among symptoms of AWS, which are related to psychopathology and would otherwise be challenging to disentangle using traditional approaches. Although the network approach has been increasingly applied to investigate the complex structure of various psychiatric disorders, including posttraumatic stress disorder, depression, and autism spectrum disorder, there is a dearth of research using this approach in the context of AWS (24–26).

To address these gaps in the literature on AWS, we conducted a proof-of-concept exploratory study. Specifically, the present study constructs a network of AWS symptoms, explores core symptoms during alcohol withdrawal, and investigates connections between different mental symptom clusters. Network analysis enhances understanding of the interactions and interconnectedness between AWS symptoms. The results could inform treatments that prevent relapse by disrupting maladaptive AWS cycles, rather than just temporarily suppressing symptoms. This could ultimately improve the management and long-term treatment of withdrawal symptoms in individuals with AUD.

## 2 Method

### 2.1 Participants

A total of 553 male AUD patients were recruited from six psychiatric hospitals in Northern China. Participants ages ranged from 20 to 67 years-old (*M* = 41.37, *SD* = 10.38). Regarding their education, over half the male patients had a junior high school education or below, and the average time in schooling was 11.25 years (SD=2.84, range = 5-17 years), see **Table 1** for more details. The inclusion criteria are listed as follows:1) Patients fulfilled the criteria of the diagnosis of alcohol use disorder based on the Diagnostic and Statistical Manual of Mental Disorders, fourth edition (DSM-IV); 2) Patients were diagnosed according to the DSM-IV diagnostic criteria by at least two qualified psychiatrists; 3) Actively drinking without extended abstinence periods in the past 3 months; 4) Currently in first 1-4 weeks of abstinence (early withdrawal stage); 5) Chinese-Han male with good reading and writing skills. Exclusion criteria were listed: 1) An individual or family history of other mental disorders; 2) Patients with severe organic disease such as CNS disease, renal, gastrointestinal system, and malignancy; 3) Any history of other substance abuse.

**Table 1:** Demographic characteristics of participants (*N* = 553).

Variable	Range/Categories	*M*	*SD*	*N*	*P*ercentage
Age	20-67	41.37	10.38		
Educational years	5-17	11.25	2.84		
Marital status					
	Married			389	70.34%
	Divorced/Separated			164	29.66%
Living status					
	Live with family			435	78.66%
	Live without family			118	21.34%

### 2.2 Procedure

After all enrolled patients provided written informed consent, the patient was then led to an assessment room under the company of the hospital psychiatrist and asked to complete psychiatric symptom assessment questionnaires in addition to the routine diagnosis process. Once the patient was completed, the psychiatrist checked to ensure that all sections of assessment questionnaires had been completed. All procedures contributing to this work conformed to the ethical standards of the relevant national and institutional human experimentation committees, as well as to the 1975 Declaration of Helsinki (revised 2008). All procedures involved were approved by the IRB of the Inner Mongolian Medical University on October 30, 2015 (YKD2015003).

### 2.3 Measurements

#### 2.3.1 Aggression

Aggression was assessed by the Buss-Perry Aggression Questionnaire (BPAQ), which is one of the most widely used self-report tools to assess aggression. The BPAQ consists of four components: anger, hostility, physical aggression and verbal aggression (27). Physical aggression and verbal aggression represent the behavioral component of aggression, anger represents the emotional component, and hostility represents the cognitive component (28, 29). The questionnaire measures in a 5-point Likert scale with higher scores reflecting more aggression. BPAQ has proven to be a reliable indicator of aggression in Chinese populations with a Cronbach’s alpha of 0.72-0.85 (30).

#### 2.3.2 Impulsivity

Impulsivity were measured by the Barratt Impulsiveness Scale (BIS) (31), which is a 30-item self-report scale with three factors:1) cognitive impulsivity, the tolerance for cognitive complexity and persistence; 2) motor impulsivity, the tendency to act on the spur of the moment; 3) non-planning impulsivity, the lack of sense of the future (32). The questionnaire measures in a 5-point Likert scale with higher scores reflecting more aggression. BIS has satisfactory reliability and validity when applied in clinical studies, with a Cronbach’s alpha of 0.83 (33).

#### 2.3.3 Negative emotions

Negative emotions were measured by Self-Rating Anxiety Scale (SAS) and Self-Rating Depression Scale (SDS). SAS and SDS are widely used questionnaires in alcohol use disorder, the Chinese version of which was used in previous studies (34). Both scales have 20 items, and all items are rated on a 4-point scale (1 = a little time to 4 = most of the time (35). The total score is multiplied by 1.25 and is then converted into a standardized score ranging from 25 to 100, higher total scores indicate more severe symptoms of depression or anxiety. The SAS and SDS has been validated have great internal consistency with a Cronbach’s alpha of 0.79 to 0.82 (36, 37).

#### 2.3.4 Self-control

Self-control was assessed using the 13-item Brief Self-Control Scale (SCS), scored on a response scale from 1 (very much) to 5 (none at all) with higher scores indicate a lower level of self-control. SCS Has been previously shown to provide a reliable and valid explanation for self-control in individuals (38).

### 2.4 Statistical analysis

R 4.2.1 software was used to construct the network of AWS. First, we calculated the partial correlations between all items to investigate the edges weight of the withdrawal network. Specifically, the edge weights are estimated using a Gaussian Graphical Model (GGM), a widespread network model (39). To reduce the likelihood of spurious connections, a Graphical Least Absolute Shrinkage and Selection Operator (GLASSO) was applied with an Extended Bayesian Information Criterion (EBIC) (40). EBIC is a common goodness-of-fit measure which select the best network from many possible networks under different “γ” (41). In present study, the ‘γ’ was set to 0.5, has been found to produce accurate network estimates, striking a good balance between sensitivity and specificity in identifying true positive margins (42). The network was visualized by the “qgraph” package, where thicker lines represented stronger relationship between symptoms, green lines represent positive relationships and red lines represents negative relationships (39). Node placement is determined by the Fruchterman-Reingold (FR) algorithm, it places nodes such that all edges have the same length, while avoiding edge crossings, and places nodes with strong average associations to the center of the graph (43). We also use the Multidimensional scaling (MDS) algorithm to place the nodes, which helps to represent complex data in a low-dimensional space, which coincides with the goal of visual presentation of complex mental networks (44).

Second, we calculated the centrality indices (node strength, closeness, betweenness, expected influence) to identify central or most important symptoms in the network via the R package “networktools”. Strength is the absolute value of the weight on the edge connected to the node. Closeness is defined as how close a node is to the average edge distance of all other nodes. Betweenness is the number of times a node is on the shortest path between any other two nodes. Expected influence is a new centrality metric proposed by Robinaugh et al., which aims to assess a node’s influence with its immediate neighbors (45). In addition to network centrality, the clustering coefficient of network nodes is also important for identifying core network importance, which is usually neglected (39, 46). The clustering coefficient can be interpreted as an indicator of the redundancy of nodes in their neighborhoods. A high clustering coefficient indicates that the neighboring nodes of the node have strong connections with each other, so removing or changing this node would not significantly affect the other nodes, which would greatly reduce the node’s importance in the network structure.

Third, we examined the stability of the edge weight and centrality indices of the network to ensure that the network is stable enough. We conducted bootstrap approach to calculate 95% confidence intervals (CIs) to assess the accuracy of the centralities and estimated the correlation stability coefficients (C S-coefficient) via the “bootnet” package in R (39).

Fourth, we used the R package “networktools” to analyze bridge symptoms reflected by bridge centrality (47), which includes bridge strength (total connectivity of nodes and other symptom cluster nodes), bridge closeness (the average distance from a node to all nodes outside of its disorder, with distance based upon the inverse of the edge weights in a weighted network) and bridge betweenness (the number of times a node lies on the shortest path between any two nodes from two distinct symptom clusters). In current study, AWS can be regarded as a collective system of 4 symptom clusters (self-control symptom clusters containing only one node were not included in the bridge symptom analysis).

## 3 Result

### 3.1 Descriptive analytics

Correlation analysis among AUD patients revealed significant relationships between demographic factors and key variables (**Table 2**). Age was positively correlated with non-planning impulsivity (*r* = 0.14, *p* < 0.01), motor impulsivity (*r* = 0.14, *p* < 0.01), cognitive impulsivity (r = 0.13, p < 0.01), verbal aggression (*r* = 0.14, *p* < 0.01), anger (*r* = 0.20, *p* < 0.001), hostility (*r* = 0.15, *p* < 0.001), anxiety (*r* = 0.14, *p* < 0.01). Educational years were negatively correlated with non-planning impulsivity (*r* = -0.27, *p* < 0.001), motor impulsivity (*r* = -0.20, *p* < 0.001), cognitive impulsivity (*r* = -0.25, *p* < 0.001), physical aggression (*r* = -0.23, *p* < 0.001), verbal aggression (*r* = -0.22, *p* < 0.001), anger (*r* = -0.23, *p* < 0.001), hostility (*r* = -0.21, *p* < 0.001), anxiety (*r* = -0.20, *p* < 0.001), and self-control (*r* = -0.10, *p* < 0.05). t-test results indicated that divorced or separated AUD patients had significantly higher levels of physical aggression compared to married individuals (*t* = 2.06, *p* = 0.04, *Cohen’s d* = 0.19), with no significant differences in other symptoms (*p* > 0.05). No significant differences were observed in AWS related symptoms based on whether AUD patients lived with family members (*p* > 0.05).

**Table 2:** Correlations of demographic factor and alcohol withdrawal syndrome.

Variables	1	2	3	4	5	6	7	8	9	10	11	12
1.Age	1											
2.Educational years	-0.44^***^	1										
3. Non-planning Impulsivity	0.14^**^	-0.27^***^	1									
4. Motor Impulsivity	0.14^**^	-0.20^***^	0.34^***^	1								
5. Cognitive Impulsivity	0.13^**^	-0.25^***^	0.77^***^	0.26^***^	1							
6. Physical Aggression	0.05	-0.23^***^	0.24^***^	0.46^***^	0.13^**^	1						
7. Verbal Aggression	0.14^**^	-0.22^***^	0.19^***^	0.50^***^	0.09^*^	0.67^***^	1					
8. Anger	0.20^***^	-0.23^***^	0.30^***^	0.62^***^	0.20^***^	0.66^***^	0.75^***^	1				
9. Hostility	0.15^***^	-0.21^***^	0.24^***^	0.57^***^	0.16^***^	0.61^***^	0.69^***^	0.67^***^	1			
10. Anxiety	0.14^**^	-0.20^***^	0.25^***^	0.38^***^	0.17^***^	0.28^***^	0.32^***^	0.40^***^	0.45^***^	1		
11. Depression	0.02	-0.04	0.14^**^	0.13^**^	0.08	0.11^**^	0.12^**^	0.19^***^	0.15^***^	0.17^***^	1	
12. Self-control	0.02	-0.10^*^	0.19^***^	0.24^***^	0.13^**^	0.29^***^	0.25^***^	0.32^***^	0.30^***^	0.14^**^	0.08	1

^*^P < 0.05, **P < 0.01, ***P < 0.001.

### 3.2 AWS network structures

**Figure 1:**
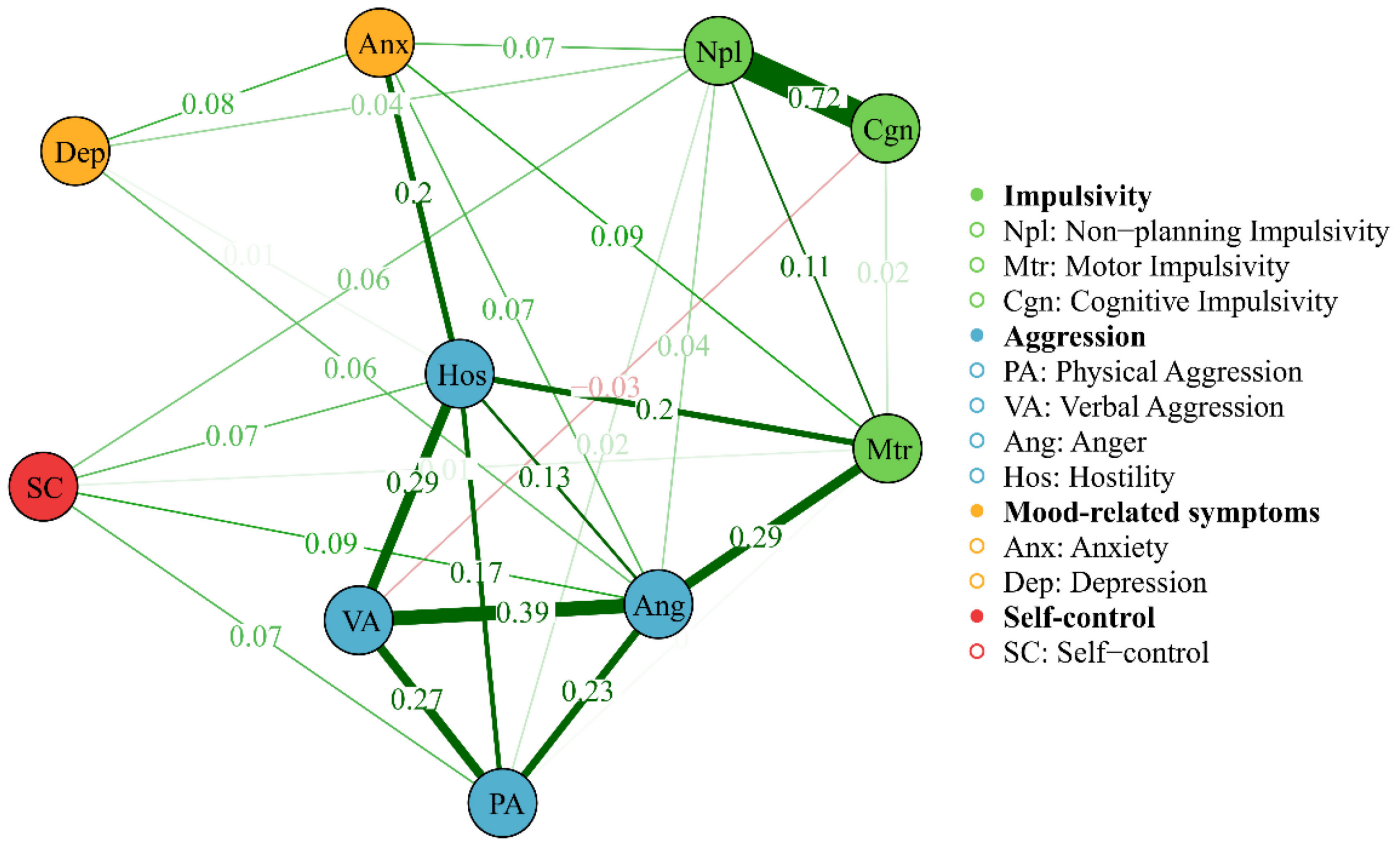
Network of AWS with FR algorithms, showing network edges and clustering structure. The thickness of the edge reflects the magnitude of the correlation, green edges represent positive correlations and red edges indicate negative correlations.

**Figure 2:**
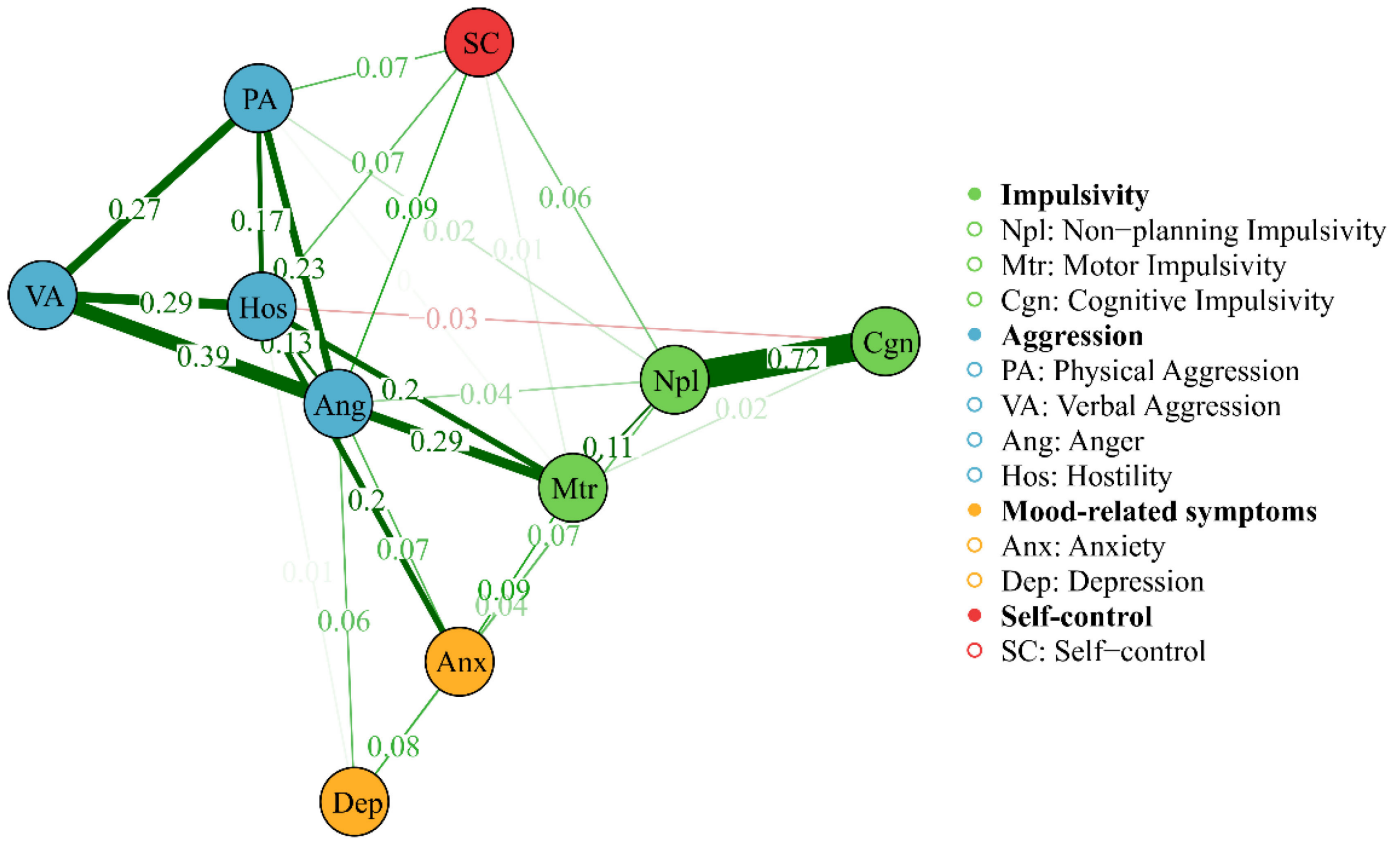
Network of AWS with MDS algorithms, showing the proximity between variables as the distance between the point in a low-dimensional space. The thickness of the edge reflects the magnitude of the correlation, green edges represent positive correlations and red edges indicate negative correlations.

**Table 3** provides the descriptive statistics and edge weight of all the AWS network nodes. **Figures 1**, **2** display the network of AWS with FR algorithms and MDS algorithms respectively. As is shown in **Figures 1**, **2**, the network of AWS consists of four symptom clusters containing ten symptom nodes, 37.8% (17/45) of network edges were set to zero, and the mean edge weight of the network was 0.08. First, the connection of network edges is analyzed within four symptom clusters. The two strongest connections were found between “non-planning impulsivity” and “cognitive impulsivity” (edge weight = 0.72) within the impulsivity symptom cluster, and between “anger” and “verbal aggression” (edge weight = 0.39) within the aggression symptom cluster. Secondly, the connection of network edges is analyzed among four symptom clusters. The two strongest connections were found between “anger” from the aggression symptom cluster and “motor impulsivity” (edge weight = 0.29) from the impulsivity symptom cluster, and between “Hostility” from the aggression symptom cluster and “anxiety” (edge weight = 0.20) from the negative emotion symptom cluster. Additionally, self-control was found to be connected with both aggressive and impulsive symptom clusters (edge weight = 0.06 to 0.09), but not with mood-related symptoms.

**Table 3:** Edge weight matrix for AWS network.

Node	M	SD	1	2	3	4	5	6	7	8	9	10
1. Non-planning Impulsivity	41.42	19.29	1.00									
2. Motor Impulsivity	33.99	18.15	0.11	1.00								
3. Cognitive Impulsivity	40.28	17.48	0.72	0.02	1.00							
4. Physical Aggression	34.70	21.09	0.02	0.00	0.00	1.00						
5. Verbal Aggression	34.11	19.90	(--)	(--)	-0.03	0.27	1.00					
6. Anger	34.67	23.27	0.04	0.29	(--)	0.23	0.39	1.00				
7. Hostility	27.17	18.90	(--)	0.20	(--)	0.17	0.29	0.13	1.00			
8. Anxiety	33.57	9.28	0.07	0.09	(--)	(--)	(--)	0.07	0.20	1.00		
9. Depression	55.16	9.28	0.04	(--)	(--)	(--)	(--)	0.06	0.01	0.08	1.00	
10. Self-control	22.68	4.06	0.06	0.01	(--)	0.07	(--)	0.09	0.07	(--)	(--)	1.00

(--) indicates that there was no connection between two nodes.

### 3.3 The core psychopathology of AWS

**Figure 3:**
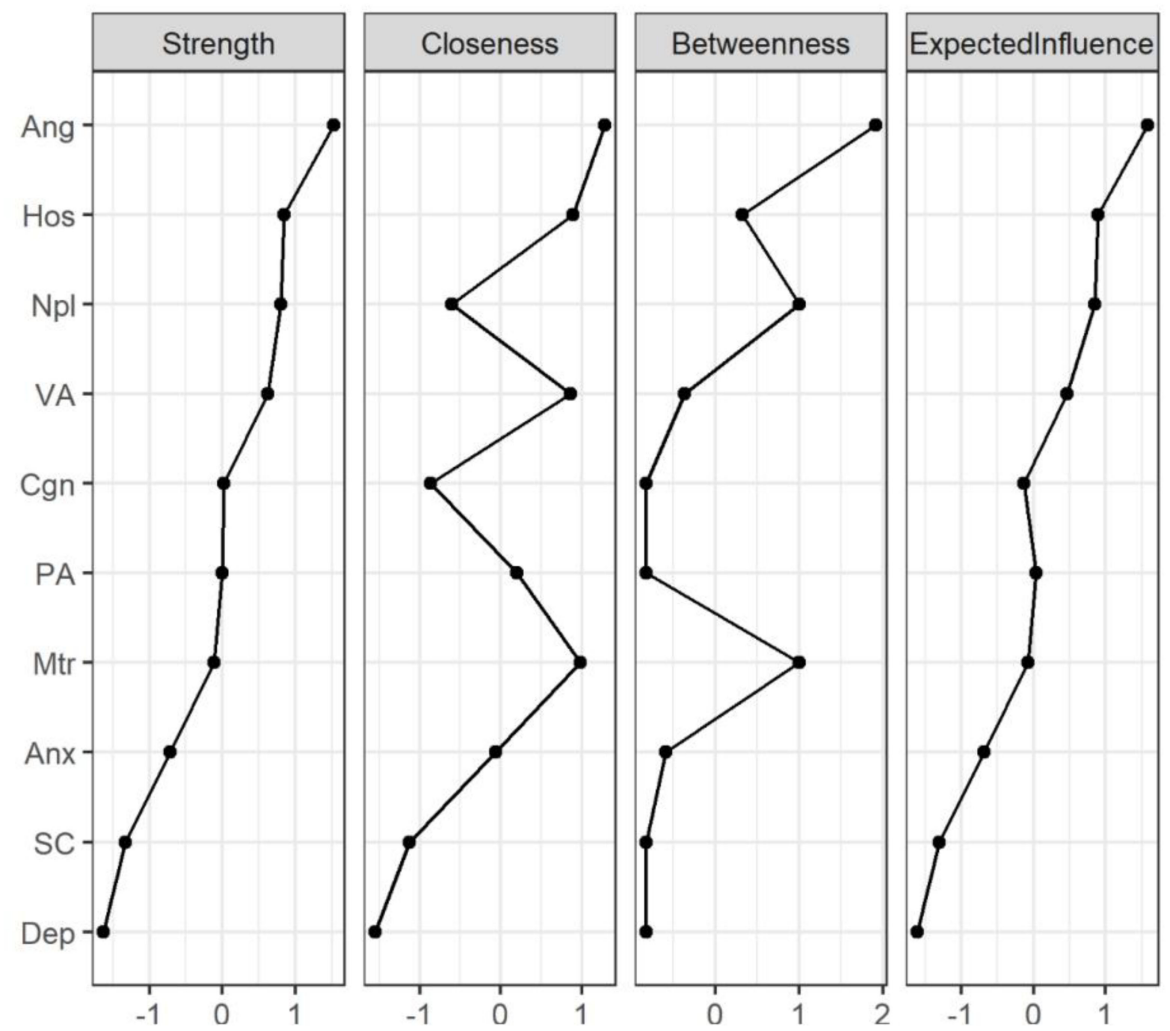
Centrality indices of network structure of AWS. The figure shows centrality measures (i.e., strength, betweenness, closeness and expected influence) of all symptoms within the network (z-scores). The full names of the abbreviations can be found in **Figure 1**. PA, Physical Aggression; VA, Verbal Aggression; Ang, Anger; Hos, Hostility; Npl, Non-planning Impulsivity; Mtr, Motor Impulsivity; Cgn, Cognitive Impulsivity; Anx, Anxiety; Dep, depression; SC, Self-control.

The network centrality indices for the AWS network are depicted in **Figure 3** and **Table 4**. First, anger showed the highest strength (Strength = 1.52), expected influence (Expected Influence = 1.58), betweenness (Betweenness = 1.92) and closeness (Closeness = 1.28) in the AWS network, it suggested that anger has the most important functional interactions in the network structure, has the shortest path connecting with other nodes and acts as a mediator associated with other nodes. Secondly, the strength and expected influence of hostility (Strength = 0.84; Expected Influence = 0.89) are second only to anger in the network structure, which indicates hostility is another key feature of alcohol withdrawal syndrome. Thirdly, non-planning impulsivity is the main feature of impulsiveness in patients with AWS, its strength (Strength = 0.80) and expected influence (Expected Influence = 0.85) are rank third in the network structure and first in the impulsive symptom cluster. Fourthly, motor impulsivity had the highest closeness (Closeness = 0.98) in the impulsive symptom cluster, suggesting that motor impulsivity is a key symptom in the association of the impulsive symptom cluster with other psychiatric symptom clusters. Finally, in negative emotion cluster, anxiety shows higher strength (Strength = -0.72), closeness (Closeness = -0.06) and expected influence (Expected Influence = -0.72) than depression, indicating anxiety is the primary negative emotion in the AWS network.

**Table 4:** Centrality index and clustering coefficient of nodes in the withdrawal symptoms network.

	Centrality Index				Clustering Coefficient
Node	Strength	Betweenness	Closeness	Expected Influence	
Npl	0.80	1.00	-0.61	0.85	0.01
Mtr	-0.12	1.00	0.98	-0.08	0.09
Cgn	0.02	-0.82	-0.87	-0.14	0.04
PA	-0.01	-0.82	0.20	0.03	0.21
VA	0.62	-0.3	0.86	0.47	0.16
Ang	1.52	1.92	1.28	1.58	0.09
Hos	0.84	0.32	0.89	0.89	0.10
Anx	-0.72	-0.59	-0.07	-0.69	0.09
Dep	-1.63	-0.82	-1.55	-1.60	0.07
SC	-1.33	-0.82	-1.13	-1.31	0.12

Npl, Non-planning Impulsivity; Mtr, Motor Impulsivity; Cgn, Cognitive Impulsivity; PA, Physical Aggression; VA, Verbal Aggression; Ang, Anger; Hos, Hostility; Anx, Anxiety; Dep, depression; SC, Self-control.

**Figure 4:**
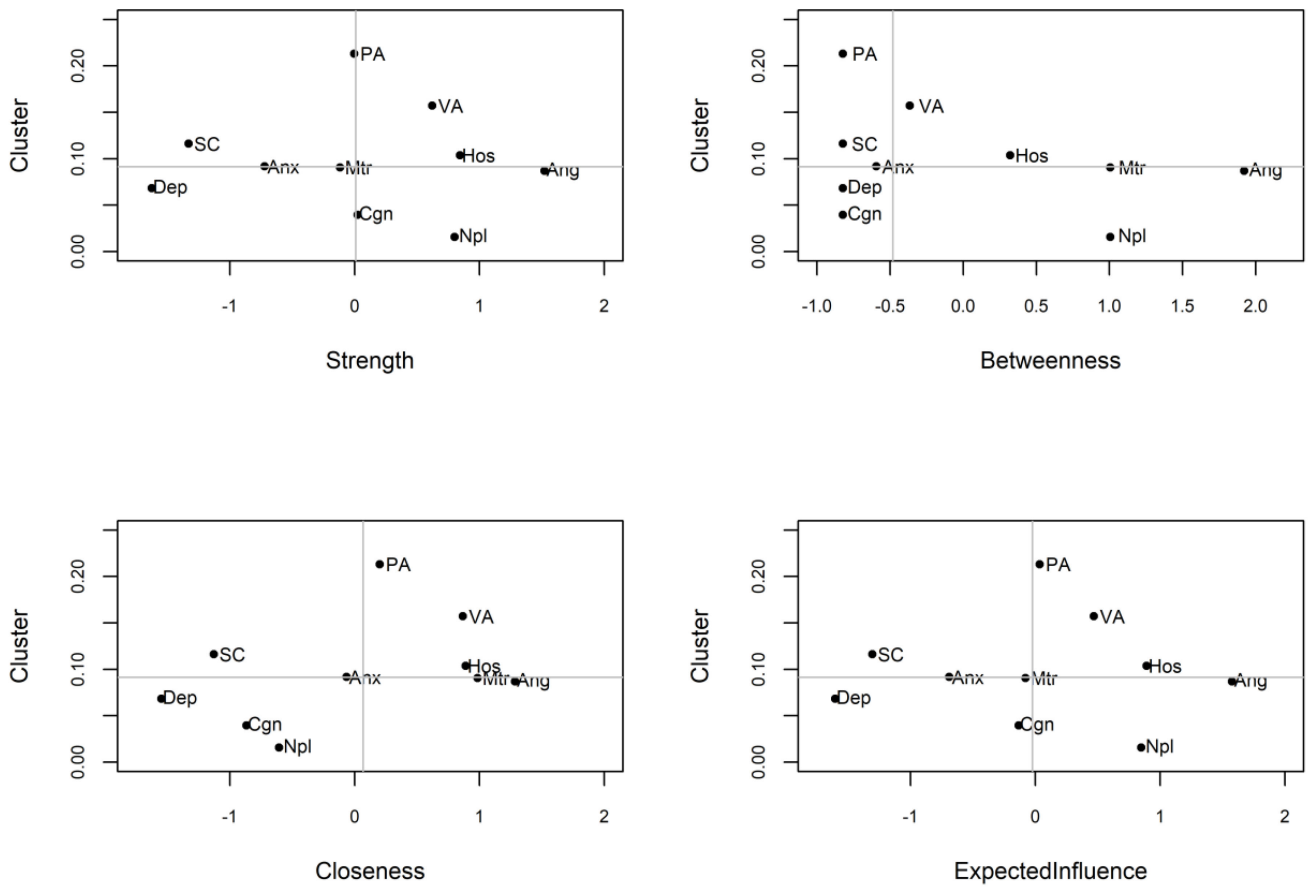
AWS network centrality index and clustering coefficient, the horizontal and vertical lines indicate the median of the clustering coefficient and centrality index respectively. PA, Physical Aggression; VA, Verbal Aggression; Ang, Anger; Hos, Hostility; Npl, Non-planning Impulsivity; Mtr, Motor Impulsivity; Cgn, Cognitive Impulsivity; Anx, Anxiety; Dep, Depression; SC, Self-control.

In order to identify the core symptoms of AWS more accurately, the testing of clustering coefficient based on the centrality index in the network was used. As shown in **Table 4** and **Figure 4**, there is no significant correlation between node centrality and clustering coefficient in the symptom network model (|*r*|s < 0.36, *p*s > 0.30). The nodes of anger, hostility and non-planning impulsiveness which are core nodes of the AWS network indicated by the centrality index analysis did not show high clustering coefficients in the clustering coefficient validation. It indicates that the centrality inflation caused by the high clustering coefficient does not exist in the process of high centrality of these three symptoms.

### 3.4 AWS network stability

Following previous studies (48, 49), the accuracy and stability of networks were tested using R package “bootnet” (50) (online **Supplementary Figures S1**, **S2** in the **Supplementary Materials**). The correlation-stability (CS) coefficient value should preferably be above 0.5 and not below 0.25 (51). In current study, edge weight was critically stable [CS (cor = 0.7) = 0.75]. Meanwhile, strength closeness and expected influence also performed perfectly [CS (cor = 0.7) = 0.75], reaching the cutoff of 0.5 and indicating that the metric was stable. In contrast, the CS coefficient of betweenness [CS (cor = 0.7) = 0.205] indicate that the betweenness of this study was not stable. Thus, strength closeness and expected influence of node were most interpretable, while betweenness can be expected to change when network is re-estimated with fewer nodes or smaller sample size.

### 3.5 Symptom connectivity loops in the AWS network

**Figure 5:**
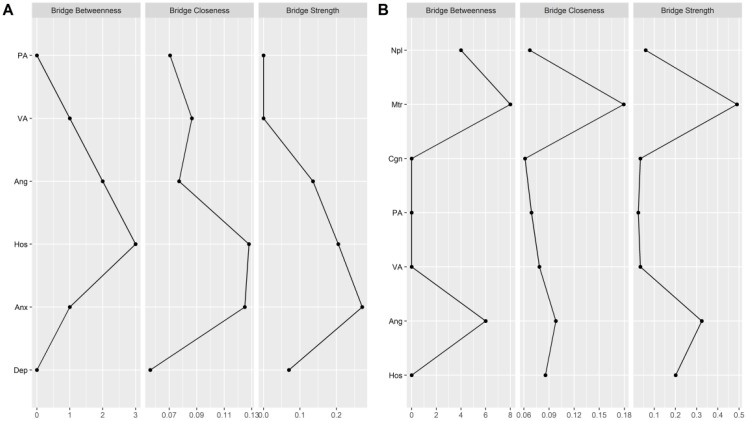
Bridge centrality indexes of network structure of AWS. **(A)** the bridge centrality indexes between aggression and mood disorder. **(B)** the bridge centrality indexes between aggression and impulsivity. PA, Physical Aggression; VA, Verbal Aggression; Ang, Anger; Hos, Hostility; Npl, Non-planning Impulsivity; Mtr, Motor Impulsivity; Cgn, Cognitive Impulsivity; Anx, Anxiety; Dep, depression; SC, Self-control.

As shown in **Figure 5**, bridge centrality indices show that there are two symptom clusters of bridge symptoms corresponding to aggression-mood disorder and aggression-impulsivity, respectively. For aggression and mood disorder, hostility (bridge strength (rank 2) = 0.20; bridge closeness (rank 1) = 0.13; bridge betweenness (rank 1) = 3) and anxiety (bridge strength (rank 1) = 0.27; bridge closeness (rank 2) = 0.12; bridge betweenness (rank 3) = 1) had the highest bridge centrality. In other words, anxiety and hostility link aggression and mood-related symptoms in alcohol withdrawal syndrome. For aggression and impulsivity, anger replaces hostility as a bridge between symptoms. Specifically, in the cluster of aggressive and impulsive symptoms, anger (bridge strength (rank 2) = 0.32; bridge closeness (rank 2) = 0.10; bridge betweenness (rank 2) = 6) and motor impulsivity (bridge strength (rank 1) = 0.49; bridge closeness (rank 1) = 0.18; bridge betweenness (rank 1) = 8) had the highest bridge centrality.

## 4 Discussion

The present study aimed to explore the psychopathological characteristics of AWS as a symptom network. Overall, the findings highlight AWS is a tightly connected and self-sustaining system of symptoms, anger and hostility were the core symptoms and the best interventions target of AWS. Additionally, impulsivity among AWS patients can be characterized by its lack of planning. In terms of mood-related symptoms anxiety is strongly interconnected with other withdrawal-related mental symptoms. Between different symptom groups, hostility-anxiety form a bridge between aggression and emotional disorder in AWS patients, while anger-motor impulsiveness forms a bridge between aggression and impulsiveness.

The descriptive results of this study further elucidate the significant correlations between demographic factors and symptoms related to AWS. Age positively correlates with several AWS-related symptoms, including impulsivity, aggression, and anxiety. This corroborates previous research indicating that older individuals with AUD experience more severe withdrawal symptoms (76). Educational years negatively correlate with AWS symptoms, suggesting a protective effect of higher education. This aligns with established literature on the inverse relationship between educational years and alcohol-related problems (77). Additionally, marital status influenced the manifestation of withdrawal symptoms. Married patients exhibited lower levels of physical aggression during withdrawal compared to non-married patients. This finding aligns with previous research indicating that married individuals often have more family responsibilities, and the social cost of physical aggression is higher, which may contribute to lower levels of aggression during withdrawal (78).

### 4.1 Symptom network of AWS

The first key finding from our network analysis of AWS is that AWS constitutes a tightly interconnected and self-sustaining system of symptoms. This network structure provides a novel perspective on the complex relationships among AWS symptoms, from NTMD. The different symptoms form a negative feedback cycle through tight interconnection and high transmission. This explains well for why AUD has a very high relapse rate (52) and withdrawal-induced psychological discomfort can persist in the absence of new stimuli (8, 9). Specifically, due to the existence of this AWS network, activation of one symptom node in daily life can be transmitted to other symptom nodes, leading to the activation of AWS network and finally leading to drinking again through a negative feedback mechanism (AWS → Drinking to cope → AUD) (53).

### 4.2 Core psychological features: anger and hostility

Network centrality index showed anger and hostility are the main psychological features of AWS, ranking highest in strength, closeness, betweenness and expected influence. This is consistent with previous findings, alcohol as a psychoactive substance can lead to emotion dysregulation and socio-cognitive impairment when consumed over a prolonged period of time or in excess (54). Anger, as a negative emotional experience, has also been shown to be a potential mediator factor in various mental disorders, including anxiety disorders, substance dependence, and post-traumatic stress disorder (55, 56). Previous studies suggest that the relationship between alcohol and anger may be mediated by the activation of glutamic acid and GABA receptors, and acute or chronic exposure to alcohol can significantly affect glutamic acid neurotransmission in the pre-frontal cortex (PFC), leading to neural adaptation, suppressing PFC activity (57, 58). The PFC, as a key area for emotion processing and social cognition, may reduce emotional control and increase anger when its activity is suppressed. Hostility, as a cognitive component, has been established as a core symptom of schizophrenia in previous network analysis studies (59), and an increase in hostility is associated with more severe positive symptoms of schizophrenia and greater use of drugs or alcohol (60). Over the past two decades, hostile cognition has been proven to be a serious consequence of alcohol use disorder and is associated with lower social functioning and poorer treatment outcomes, indicating its crucial role in alcohol-related illnesses (61–63). This finding also supports the “hostile attribution bias model” of alcohol, which suggests that alcohol affects the brain’s social-cognitive processes, leading to increased hostile cognition and impaired social functioning (63). The results suggest that anger and hostility play important roles in the network of alcohol withdrawal syndrome, highlighting the key role of emotional regulation difficulties and hostile cognition in alcohol withdrawal syndrome. Meanwhile, these two symptoms (hostility and anger) may also be appropriate intervention targets to break the self-sustaining system of alcohol use disorder.

### 4.3 Impulsiveness in AWS patients

Non-planning impulsiveness was identified as the primary characteristic of AWS patients in terms of impulsiveness. Impulsiveness is a multi-dimensional structure, including attention, behavior, and cognitive components. This study also divided impulsiveness into three levels: non-planning impulsiveness, cognitive impulsiveness, and motor impulsiveness (31). Non-planning impulsiveness had the highest strength and expected influence in the entire withdrawal symptom network and was the main characteristic of impulsiveness. Previous research has also indicated that long-term alcohol use by patients with alcohol use disorder can affect the central nervous system, leading to structural and functional frontal lobe deficits. Alcohol dependent patients can only control and regulate emotions and behaviors with limited resources (64, 65), when faced with high-pressure or challenging events, these individuals have difficulty regulating impulsive behavior or decisions, resulting in unplanned reckless behavior.

### 4.4 Bridge analysis of AWS network

Bridge analysis revealed that anger is the connecting node between aggression and impulsiveness, further emphasizing the relationship between a patient’s ability to regulate emotions and impulsiveness. Ineffective or low-effective emotional regulation strategies may also promote goal-directed impulsive behavior without planning. In a study utilizing cognitive-behavioral measures, Tsukue et al. (66) reported a significant correlation between more frequent impulsive choices (as measured by the delay discounting paradigm) and higher sensitivity to unfairness in patients diagnosed with alcohol use disorder (66). This latter phenomenon may be interpreted as an emotion regulation strategy (65). That can be assumed that impulsiveness is closely related to poor emotional regulation strategies or coping mechanisms during stress-inducing situations of alcohol withdrawal. Of course, This cross-sectional study does not infer any causality, but rather focuses on analyzing a specific path or circuit, where emotional disturbance may be a trigger for impulsiveness and vice versa in alcohol withdrawal. As a result, these symptoms enter a vicious cycle, causing the maintenance of alcohol withdrawal symptoms in the long term, which in turn leads to recurrent alcohol consumption (negative reinforcement) and perpetuation of the cycle.

Bridge analysis has also highlighted the notion of hostility as a bridge node connecting aggression and anxiety. Anxiety has long been linked to mental illness and plays an important role in aggression-related behaviors associated with mental illness (66), yet most models of aggression development fail to consider the role of anxiety, which is also overlooked in clinical interventions for comorbidity of aggression-anxiety (67). Moreover, hostility as a cognitive-level aggression coupled with anxiety revealed the correlation between cognitive bias and anxiety in alcohol-dependent patients during withdrawal. The abnormal combination of hostility and anxiety emerges as a response to seemingly unrelated but potentially threatening situations, which may be particularly evident in those with mental disorders. Stompe et al.’s empirical study of the dreams of schizophrenic patients found that they experienced themselves more frequently as victims of aggression from the outside world in their dreams, which also corresponded to a high degree of threat anxiety (injury) experienced in the dream, but value anxiety (guilt and separation) was less frequent in schizophrenic patients’ dreams (68). Clinical psychology also points out that hostility, as a powerful social signal, is closely related to social anxiety (69, 70). Attention studies of cognitive bias towards hostility focus primarily on the processing tendency of threatening cues in the environment, while the cognitive model of social anxiety likewise emphasizes the problem of attention bias, with extensive processing or cognitive bias towards threatening cues also promoting or maintaining social anxiety (71). Studies on brain activation of human faces in social anxiety show that the amygdala complex is related to the processing of threatening cues, and that when faced with threatening faces, the metabolic response of these nuclei increases in people with social anxiety (70). These results suggest that hostility and anxiety may be the result of neurological dysfunction during alcohol withdrawal, and that anxiety during withdrawal may have more to do with cognitive biases (hostility) than simply emotional regulation problems.

## 5 Conclusion

To summarize, this research constructed a network of AWS from the perspective of network theory of mental disorders. In the network of AWS, different withdrawal symptoms are closely related, and AWS can be maintained over a long period of time through the interaction of symptoms. Anxiety and hostility are the core psychiatric symptoms of patients with AWS and may be the best intervention points to disrupt the self-sustaining system of AUD. Impulsiveness is the main feature of the withdrawal period; in terms of emotional disorders, anxiety is highly associated with other groups of psychiatric symptoms. In addition, hostility and anxiety, anger and impulsiveness linked to different clusters of symptoms through their respective symptom cycles, suggesting that different mechanisms and neurobiological bases may be involved in withdrawal symptoms, which play a similar role in withdrawal syndrome.

## 6 Limitations

Our study had certain limitations that should not be overlooked, and it provides ideas for future research. First, the current study is a cross-sectional design, lacking longitudinal data to confirm the effectiveness of the AWS clinical intervention targets (72). This limitation means we cannot establish causal relationships or track changes over time. Future work should seek to test these intervention targets over multiple time points of the recovery process before as well as after treatment to establish whether the present simulation results can be confirmed. Second, we did not include sociodemographic factors and motivation-to-change as nodes in our network, which are important to sustain the system of withdrawal syndrome (73, 74). The exclusion of these factors may limit our understanding of the full complexity of AWS and its underlying mechanisms. Third, this study examined only AWS-related emotional and behavioral symptoms, lacking relevant physiological data (75). This omission means we could not explore the physiological changes that accompany AWS, which could provide a more comprehensive understanding of the syndrome. Finally, this study sample was restricted to male patients only. The decision to include only male participants was based on the significantly higher prevalence of AUD among men compared to women, which allowed for more efficient recruitment of a clinically relevant sample. According to the National Institute on Alcohol Abuse and Alcoholism (NIAAA), the prevalence of AUD is approximately 12.4% in men and 4.9% in women in the United States (79). Additionally, focusing on a single gender helped to reduce potential confounding variables related to gender differences in alcohol metabolism and sociocultural factors influencing alcohol use and withdrawal symptoms. Given the known sex differences in substance use disorders, this limits the generalizability of our findings to female alcohol use disorder populations. Future studies should aim to include a more diverse participant pool, encompassing both genders, to provide insights into gender-specific differences in AUD and withdrawal syndrome.

## Data Availability

The raw data supporting the conclusions of this article will be made available by the authors, without undue reservation.
