# Supplementary material for: Exploring core symptoms of alcohol withdrawal syndrome in alcohol use disorder patients: a network analysis approach
Source: Front Psychiatry. 2024 Aug 29;15:1320248. doi: 10.3389/fpsyt.2024.1320248 (PMC11390437; doi:10.3389/fpsyt.2024.1320248)
Supplement: Supplementary file 1 [file DataSheet1.docx]

**Supplementary Materials**


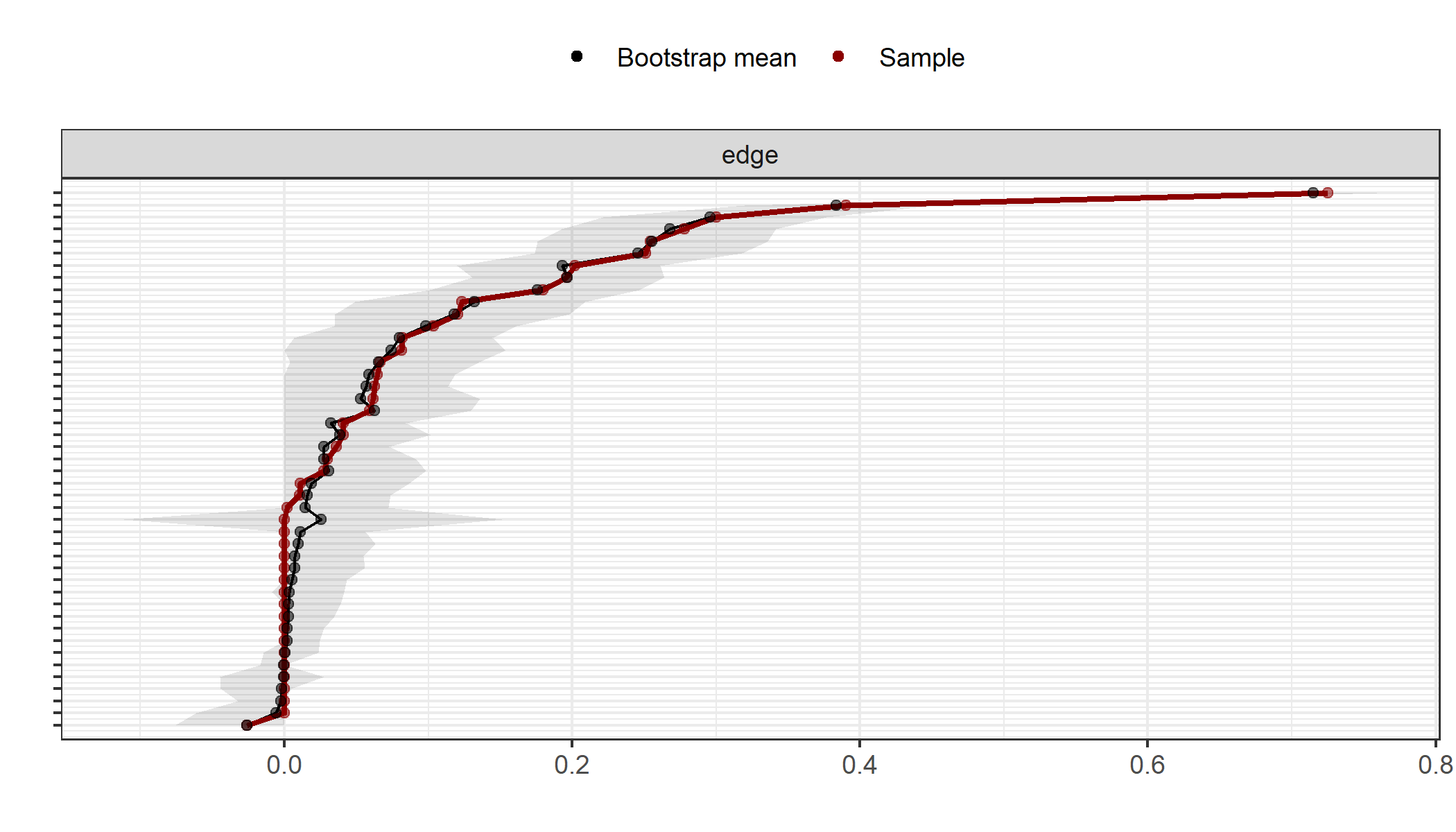
 Supplementary Figure S1. Edge stability test bootstrap estimates 95% confidence interval for edge weights. Red represents the weight of the network connection of symptoms in this study, black represents the average weight calculated by Bootstrap, and the gray area represents the 95% confidence interval.


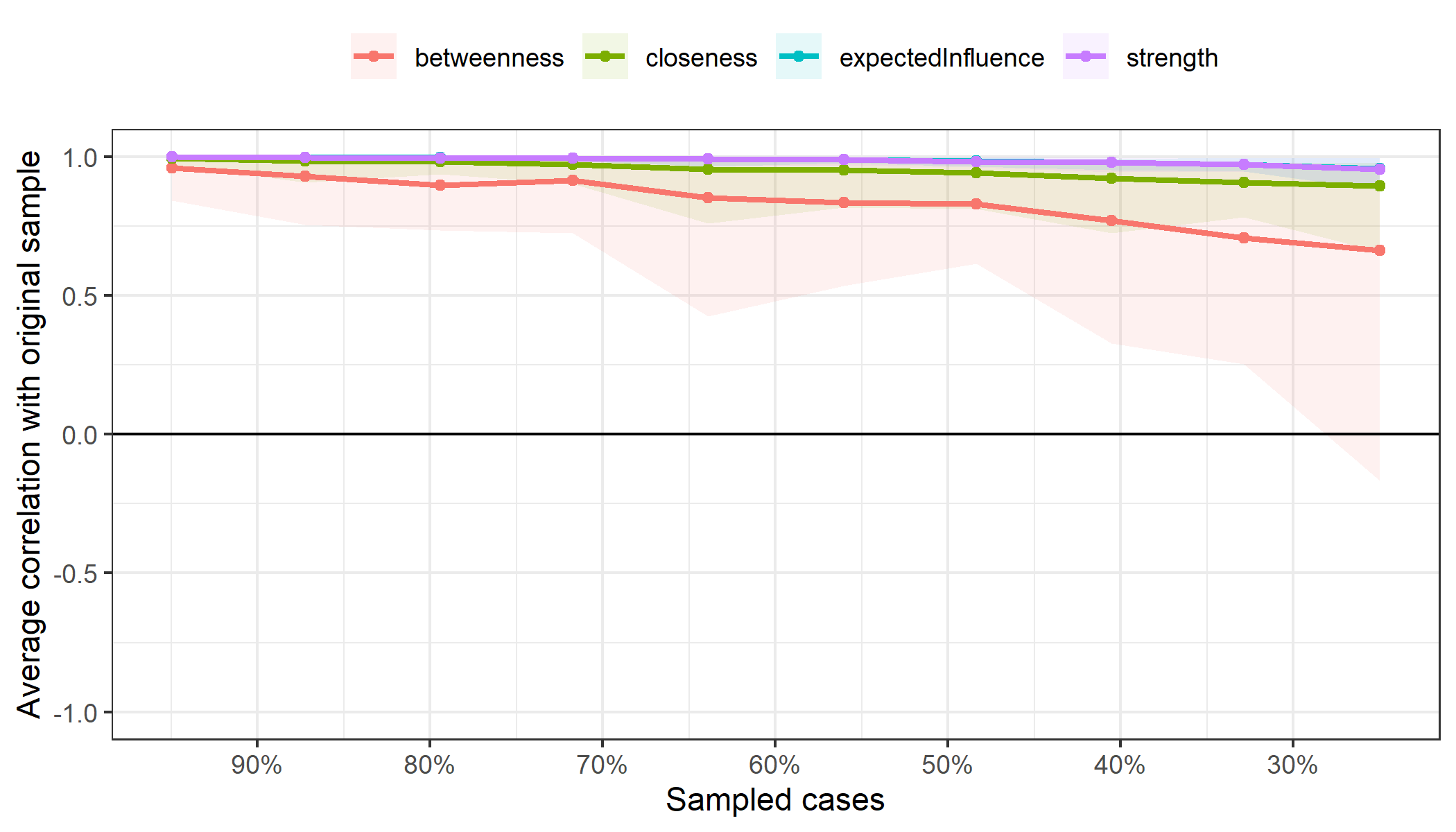
 Supplementary Figure S2. Edge stability test bootstrap estimates 95% confidence interval for centrality indices. The abscissa indicates the proportion of included samples, and the ordinate indicates the correlation between the centrality indicators of the symptom network model nodes and the centrality indicators after changing the proportion of included samples.
